# Identification of replication-dependent and replication-independent linker histone complexes: Tpr specifically promotes replication-dependent linker histone stability

**DOI:** 10.1186/s12858-016-0074-9

**Published:** 2016-10-01

**Authors:** Pei Zhang, Owen E. Branson, Michael A. Freitas, Mark R. Parthun

**Affiliations:** 1 Department of Biological Chemistry and Pharmacology, The Ohio State University, Columbus, OH 43210 USA; 2 Department of Cancer Biology and Genetics, The Ohio State University, Columbus, OH 43210 USA

**Keywords:** Chromatin, Histone, Histone chaperone, Linker histone, H1, Tpr

## Abstract

**Background:**

There are 11 variants of linker histone H1 in mammalian cells. Beyond their shared abilities to stabilize and condense chromatin, the H1 variants have been found to have non-redundant functions, the mechanisms of which are not fully understood. Like core histones, there are both replication-dependent and replication-independent linker histone variants. The histone chaperones and other factors that regulate linker histone dynamics in the cell are largely unknown. In particular, it is not known whether replication-dependent and replication-independent linker histones interact with distinct or common sets of proteins. To better understand linker histone dynamics and assembly, we used chromatography and mass spectrometry approaches to identify proteins that are associated with replication-dependent and replication-independent H1 variants. We then used a variety of in vivo analyses to validate the functional relevance of identified interactions.

**Results:**

We identified proteins that bind to all linker histone variants and proteins that are specific for only one class of variant. The factors identified include histone chaperones, transcriptional regulators, RNA binding proteins and ribosomal proteins. The nuclear pore complex protein Tpr, which was found to associate with only replication-dependent linker histones, specifically promoted their stability.

**Conclusion:**

Replication-dependent and replication-independent linker histone variants can interact with both common and distinct sets of proteins. Some of these factors are likely to function as histone chaperones while others may suggest novel links between linker histones and RNA metabolism. The nuclear pore complex protein Tpr specifically interacts with histone H1.1 and H1.2 but not H1x and can regulate the stability of these replication-dependent linker histones.

**Electronic supplementary material:**

The online version of this article (doi:10.1186/s12858-016-0074-9) contains supplementary material, which is available to authorized users.

## Background

The basic repeating structural unit of eukaryotic chromatin is the nucleosome core particle (NCP), which is a structured package of 147 bps of DNA and a histone octamer consisting of 2 copies of each core histone: H2A, H2B, H3, and H4. There are also linker histones, H1, flanking NCPs and sealing the structure with about 20 bps of additional DNA. The involvement of H1 is crucial for the formation and stabilization of chromatin structures and for the regulation of gene expression [1, 2].

There are 11 variants of linker histone H1 in mammalian cells: somatic replication-dependent variants (H1.1 to H1.5), somatic replication-independent variants (H1.0, H1x), and germ cell specific variants (H1t, H1T2m and HILS1 for testicular cells, and H1oo for oocytes) [3]. The replication-dependent variants are typically expressed during S phase and are incorporated during into chromatin during DNA replication, while the replication-independent variants are expressed throughout the cell cycle and can be incorporated into chromatin outside of S phase. The sequences of these H1 variants vary greatly in their C-terminal domains. The individual functions of H1 variants are not fully understood. It was believed that H1 served as a global gene regulator by binding to chromatin non-specifically. However, although partially redundant in function, there is evidence indicating H1 variants have distinct roles in gene regulation and development. While deleting a single H1 variant did not lead to any observable phenotype, the H1.2/H1.3/H1.4 triple knockout mouse exhibited developmental defects and embryonic lethality [4]. Microarray experiments showed that knockdown of each H1 variant altered a different subset of genes [5]. The expression levels and activities of the H1 variants were also found to be highly regulated during cell differentiation and tumorigenesis [6]. These observations suggest the hypothesis that each H1 variant has its individual function in the cells in addition to their roles as global chromatin modifiers.

Studies on core histones indicate that replication-dependent and replication-independent histone variants can be involved in distinct protein complexes and assembly pathways. For example, histone H3.1 is assembled into chromatin in coordination with DNA replication during S phage, while another variant, H3.3, is exchanged throughout the cell cycle. The dynamics of H3.1 and H3.3 are mediated by distinct protein complexes that contain different chaperones for each of the H3 variants. While H3.1 and H3.3 complexes have some shared histone associated proteins, such as NASP, ASF1A, ASF1B, HAT1, and importin 4, there are also exclusive histone binding partners in each complex. All three subunits of histone chaperone CAF-1 are only found in the H3.1 complex, while another histone chaperone, HIRA, is only found in the H3.3 complex [7, 8]. CAF-1 and HIRA then dictate the replication-coupled and replication-independent assembly of these H3 variants.

Currently it is not known whether the dynamics of replication-dependent and -independent variants of H1 are also regulated by distinct chaperones in a manner similar to H3.1 and H3.3 complexes. Previous studies proposed several linker histone chaperone candidates: Nucleosome Assembly Protein 1(NAP1) [9], Nuclear Autoantigenic Sperm Protein (NASP) [10], Nucleophosmin (NPM1) [11], Prothymosin α (ProTα) [12], and Template Activating Factor-I (TAF-1, also known as protein SET) [13]. The roles of these interactions between histone H1 and linker histone chaperones in H1 storage, transport, chromatin assembly and disassembly are not fully understood. How the linker histone chaperones interact with each H1 variant also remains to be determined. To better understand H1 variant - protein interactions, we purified non-chromatin associated protein complexes containing 6 × His-tagged H1 variants expressed in Tetracycline-inducible U2OS cell lines using column chromatography. Proteins associated with each H1 variant were identified by mass spectrometry analysis. We found replication-dependent and replication-independent H1 variants had distinct binding partners. For example, one protein bound to replication-dependent H1.1 and H1.2, nucleoprotein Tpr, was not found in the protein complex containing replication-independent H1x. Tpr knockdown leads to decreased levels of H1.1 and H1.2, but does not affect H1x levels. These findings suggest that association with variant-specific binding partners may regulate linker histone dynamics.

## Results

### Generation of inducible U2OS cell lines expressing 6 × His-tagged H1 variants

**Fig. 1:**
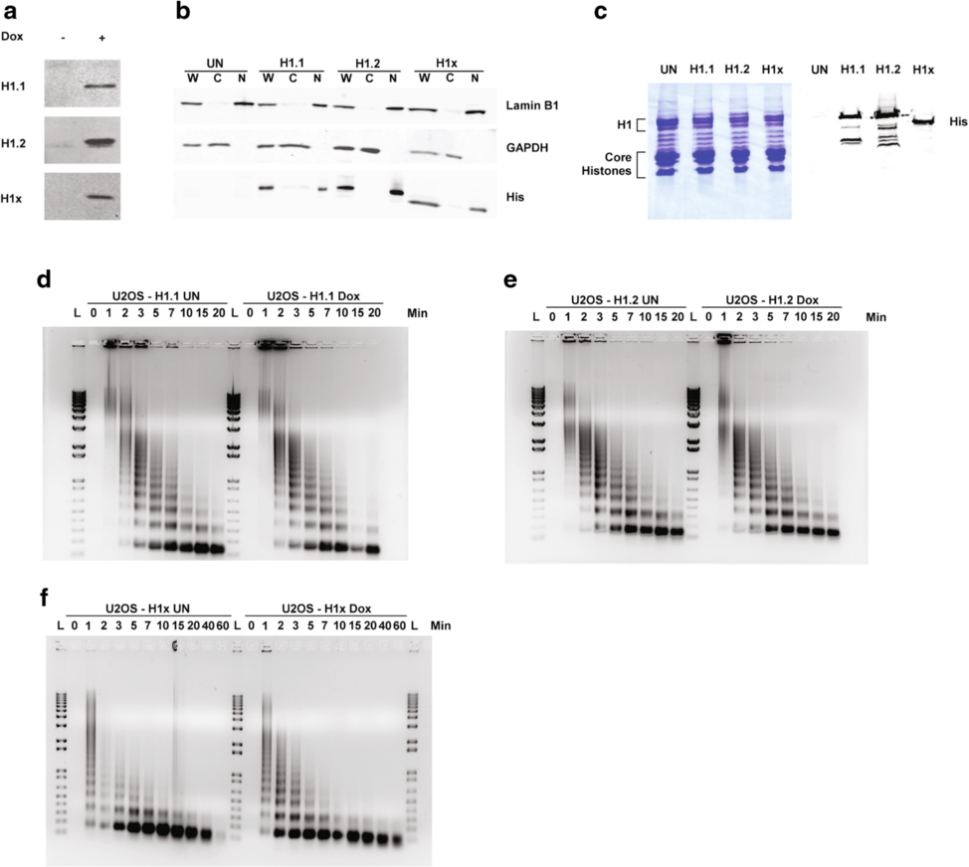
Over-expression of 6 × His-tagged H1 Variants Does Not Alter Global Chromatin Structure. **a** U2OS Tet/On cells were transfected with plasmids encoding 6 × His-tagged human histone H1 variants. These cells were incubated 72 h with or without doxycycline before extracted. Whole cell extracts were resolved by SDS-PAGE gel, and immunoblotted with anti-His antibodies. **b** Untransfected (UN) U2OS Tet/On cells or U2OS Tet/On cells expressing H1 variants (H1.1, H1.2 and H1x) were incubated with doxycycline for 72 h. Whole cell, cytosolic and nuclear extracts of these cells were resolved by SDS-PAGE gels and visualized by western blotting with antibodies targeting proteins indicated on the right. **c** Total histones were extracted from untransfected (UN) U2OS Tet/On cells or Doxycycline-induced U2OS cells expressing 6 × His-tagged H1 variants (H1.1, H1.2 and H1x) using acid precipitation method. 30 μg of each sample was resolved by SDS-PAGE gel, and visualized by coomassie staining or immunoblotting with anti-His antibodies. **d**–**f** Nuclei from uninduced (UN) or Doxycycline induced (Dox) U2OS cells transfected with vectors expressing H1.1 (**d**) , H1.2 (**e**) or H1x (**f**) were digested with 0.2 U/ml (Sigma units) of MNase at 37 °C for various lengths of time, then quenched with EDTA. Digested DNA samples were purified using phenol extraction and ethanol precipitation, and resolved on 1 % agarose gel with EtBr staining

To purify H1 variants and their associated proteins for identification, we generated tetracycline-inducible U2OS cell lines over-expressing 6 × His-tagged H1 variants H1.1, H1.2 or H1x by transfecting U2OS cells engineered for tetracycline-inducible expression with pT-Rex-DEST31 plasmids carrying the corresponding H1 variant sequences (Fig. 1a). Cellular fractionation revealed that the vast majority of 6 × His-tagged linker histones were in the nuclear fraction (Fig. 1b), indicating the exogenous H1 proteins were properly localized. Since histone proteins are highly basic, excess accumulation of histones could disrupt normal chromatin structures and cause cytotoxicity. To verify that our over-expression of H1 variants did not disrupt normal cell functions, we isolated total histones from uninduced and doxycycline-induced U2OS cells. Figure 1c shows a Coomassie stained gel of uninduced and induced cells expressing histone H1.1, H1.2 and H1x. It is clear that H1 induction does not result in an overall increase in linker histone abundance. MNase digestion assays further confirmed that over-expression of 6 × His-tagged H1 variants did not significantly alter nucleosome organization or repeat length (compare time points from uninduced samples to the Dox-induced samples in Fig. 1d–f). Thus we conclude that these cell lines are suitable as the source for H1-containing protein complex purification.

### Purification and identification of H1 complexes

**Fig. 2:**
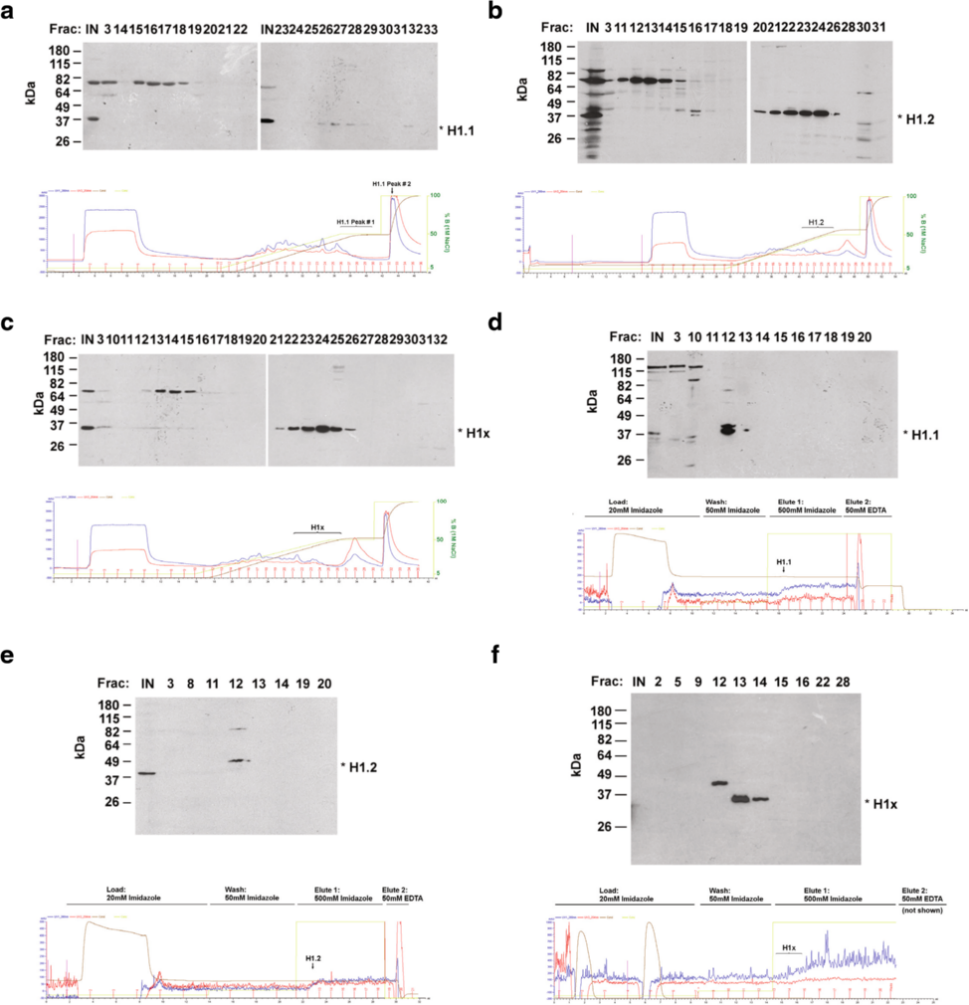
Purification of H1.1, H1.2 and H1x Complexes. **a**–**c** Soluble whole cell extracts of U2OS cells expressing 6 × His - tagged H1.1 (**a**), H1.2 (**b**) or H1x (**c**) were purified on a Mono Q column. Top panel: 1 ml fractions were collected, resolved by SDS-PAGE gel, and immunoblotted with anti-His antibodies. Bottom panel: chromatogram showing chromatography conditions used. **d**–**f** Peaks containing 6 × His – tagged H1.1 (**d**), H1.2 (**e**) or H1x (**f**) from Mono Q column were pooled and applied to a nickel column in loading buffer containing 20 mM imidazole, washed with 50 mM imidazole and then eluted with 500 mM imidazole. Top panel: 1 ml fractions were collected, resolved by SDS-PAGE gel, and visualized by immunoblotting with anti-His antibodies. Bottom panel: chromatogram showing chromatography conditions used

To analyze proteins associating with the soluble pool of the H1 variants, we purified H1.1, H1.2 and H1x from U2OS whole cell extracts by sequential chromatography with an anion exchange column (Mono Q) and an affinity column (nickel column). The specific order of the chromatography was used to optimize the concentration of proteins prior to mass spec analysis. Interestingly, H1.1 eluted in two separate peaks on the Mono Q column, while H1.2 and H1x both eluted in only one peak (Fig. 2a–c). Each peak eluting from the Mono Q column was collected and pooled separately, and chromatographed on a nickel chelate column (Fig. 2d–f, data not shown for the second peak of H1.1). We performed mock purification from whole cell extracts of untransfected U2OS Tet/On cells, which did not express 6 × His-tagged proteins, to serve as negative controls. For the mock purifications, extracts were resolved on a Mono Q column and the fractions containing an equivalent salt concentration as the peak of the H1-containing fractions was then resolved on a nickel chelating column.

**Fig. 3:**
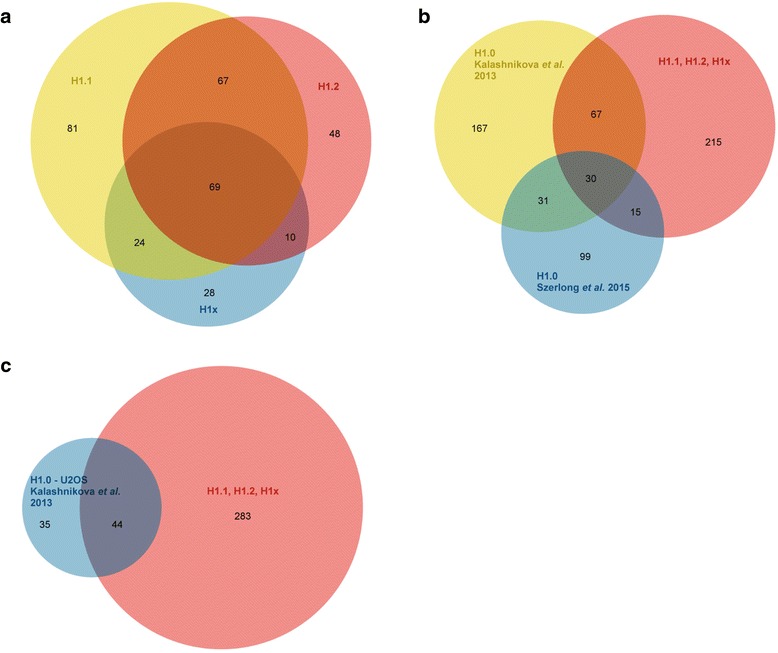
Graphical representation of identified linker histone binding proteins. **a** Venn diagram of the distribution of proteins co-purifying with histones H1.1, H1.2 and H1x (as indicated). **b** Venn diagram of the distribution of linker histones binding proteins identified in the current study (H1.1, H1.2, H1x and the studies of Kalashnikova et al. 2013 and Szerlong et al. 2015 (as indicated) [14, 15]. **c** Venn diagram of the overlap of liner histone binding proteins identified from U2OS cells in the current study and the study of Kalashnikove et al. 2013 (as indicated) [15]

Peak samples eluting from the nickel chelate column containing each histone variant, as well as the comparable negative control fraction, were analyzed in duplicate by mass spectrometry. A complete list of the proteins identified, as well as the number of unique peptides observed in each analysis, is listed in Additional file 1: Table S1. The distribution of specificities of the identified linker histone binding proteins for H1.1, H1.2 and H1x is represented by a Venn diagram in Fig. 3a. Approximately half of the proteins identified interact with more than one linker histone variant while half are specific for a single H1 species.

The protein list contains a large number of ribosomal proteins. Several recent proteomic studies have also found that linker histones bind to ribosomes and this interaction has been implicated in transcriptional repression [14–17]. Recent proteomic studies of linker histone binding proteins have used a fundamentally different approach where a histone H1 variant was immobilized and incubated with nuclear or nucleolar extracts [14, 15]. In addition to these differences in experimental approach, these studies used a different linker histone variant than those used in the current study. Despite these differences, there is significant overlap in the H1 interacting proteins identified. As seen in Fig. 3b, there is approximately 30 % overlap in the linker histone binding proteins identified in the current study and in the previous studies. In addition, if only the linker histone binding proteins identified from the same cell line are considered (U2OS), the current study identified more than half of the linker histone binding proteins found by Kalashnikova and colleagues (Fig. 3c).

Table 1 lists all of the non-ribosomal proteins found to co-purify with the linker histones that were detected by at least 5 peptides in both of the duplicate samples (average number of peptides listed). These proteins are divided into 6 groups based on their pattern of interaction. Group 1 consists of the proteins that co-purified with all three linker histones. Group 2 proteins co-purified with the replication-dependent H1.1 and H1.2 but not with the replication-independent H1x. Group 3, group 4 and group 5 proteins were specifically associated with a single histone H1 variant (H1.1, H1.2 and H1x, respectively) and, as expected, each of these groups contained the corresponding H1 variant (proteins specifically identified in H1.1 peak 2 are indicated by an asterisk). Group 6 proteins were associated with both histone H1.1 and H1x.

**Table 1:**
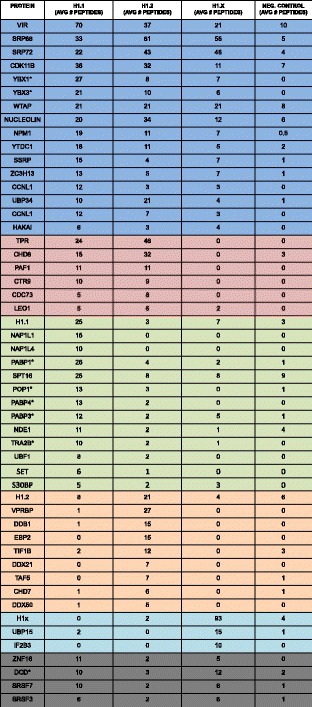
Linker histone binding proteins

Table lists the selected proteins identified by mass spectrometry associated with the linker histones H1.1, H1.2 and H1.X (color coded as indicated). Table lists the average number of peptides identified from each linker histone purification. The averages are derived from 2 to 4 replicates. * denotes proteins identified in histone H1.1 Mono-Q peak 2.  = ALL H1’s (GROUP 1),  = H1.1 AND H1.2 (GROUP 2),  = H1.1 (GROUP 3),  = H1.2 (GROUP 4),  = H1.X (GROUP 5),  = H1.1 AND H1.X (GROUP 6)

The group 1 proteins included two proteins that had previously been identified as histone chaperones, nucleolin and nucleophosmin (NPM1) [18]. Nucleolin and nucleophosmin are broad specificity histone binding proteins and have been found to associate with both core and linker histones [11, 19–22]. Group 1 also included the SSRP subunit of the FACT complex, which also has been shown to interact with both core and linker histones [15, 23]. The second subunit of FACT, Spt16, was identified as specifically interacting with histone H1.1. However, this classification was due to a high level of Spt16 peptides in the negative control sample. Therefore, it is likely that both subunits of FACT are bound to all three of the linker histone variants tested.

Group 1 also included SRP68 and SRP72. Components of the signal recognition particle, SRP68 and SRP72 have also been shown to form a heterodimeric complex distinct from the signal recognition particle. In addition to its role in translation, the SRP68/72 complex was also found to bind histone H4 molecules that contain arginine 3 methylation [24].

YBX1 and YBX3 also co-purified with all three linker histones. Y box-binding proteins bind to Y box consensus promoter elements and are involved in mRNA processing [25]. YBX1 was found to be recruited together with PURα and histone H1.2 to the p53 target gene *Bax* in order to repress p53-induced transcription [26]. Our data indicates that YBX1 not only associates with H1 variant H1.2, but with H1.1 and H1x as well. Interestingly, these Y box proteins were only detected in the H1.1 complex that corresponded to the second peak of H1.1 on the Mono Q column.

Five of the group 1 proteins are linked to N^6^-methyladenosine modification of mRNA. VIR (virilizer homolog), WTAP (Wilms Tumor Associated Protein), ZC3H13 and Hakai are all components of the WTAP complex that serves to target the METTL3 and METTL14 methyltransferases to their substrate [27–30]. In addition, YTDC1 is a YTH domain protein that can function as a reader of N^6^-methyladenosine [31–34].

The remaining group 1 proteins include a cyclin/cdk complex; CCNL1 and CDK11b. Linker histones are highly phosphorylated and often used as non-specific substrates in kinase assays. In fact, CDK11b has been shown to be able to phosphorylate histone H1 in vitro [35]. The observation that the CCNL1/CDK11b complex can be purified in association with linker histones suggests that H1s may be a specific substrate of this kinase complex. Finally, all three H1 variants associate with the ubiquitin hydrolase UBP34.

The group 2 proteins bind specifically to the replication-dependent H1 variants H1.1 and H1.2 but don’t form a complex with the replication-independent variant H1x. The group 2 proteins include 4 subunits of the PAF1 complex, PAF1, CTR9, CDC73 and LEO1 [36]. The specificity of the interaction between the PAF1 complex and H1.1 and H1.2 is consistent with a recent study that showed that PAF1 co-purified with epitope tagged H1.1 and H1.2 but not with the other replication-dependent H1 variants H1.3, H1.4, H1.5 or with the replication-independent variant H1.0. The association of the PAF1 complex with H1.1 and H1.2 was shown to function with Cul4A in transcription-associated ubiquitylation [37].

CHD8 has previously been shown to function in transcriptional repression of p53 and β-catenin target genes through the recruitment of histone H1 [38, 39]. The proteomic data presented here suggests that the interaction between CHD8 and linker histones is variant specific.

The nuclear pore complex protein Tpr was also found to be replication-coupled H1 variant specific. Tpr (translocated promoter region) is a component of the nuclear pore complex (NPC), forming fibrous structures that extend into the nuclear interior [40]. Tpr is required for establishing heterochromatin exclusion zones in the vicinity of NPCs [41]. In addition to its roles in NPC architecture, Tpr is also involved in mRNA, unspliced RNA and nuclear protein export [42–44]. Depletion of Tpr induces nuclear accumulation of p53, and facilitates autophagy [45].

Proteins that specifically co-purified with histone H1.1 (group 3) included several known histone chaperones. NapP1L1 and SET were recently shown to bind histone H1.0 in vitro [16, 46]. NAP1L4 has not previously been demonstrated to interact with linker histones.

Group 3 contained several proteins involved in RNA metabolism. These included three poly A binding proteins, PABP1, PABP3 and PABP4, as well as the RNaseP subunit POP1 [47, 48]. In addition, proteins involved in transcriptional regulation, NDE1, UBF1 and S30BP were also found to be specific for histone H1.1.

H1.2 specific binding partners (group 4) included DNA damage-binding protein 1 (DDB1) and protein VPRBP (also known as DDB1-CUL4-associated factor 1, DCAF1). DDB1 and VPRBP are both members of an E3 ubiquitin-protein ligase complex, named CUL4A-RBX1-DDB1-DCAF1/VPRBP complex, which is responsible for methylation-dependent ubiquitylation [49]. VPRBP and DDB1 were recently shown to co-purify with both H1.1 and H1.2 along with Cul4A and the PAF1 complex [37]. The distinct properties of the PAF1 and Cul4A complex subunits in the U2OS cells suggest that the specificity of the interaction between the Cul4A complex and linker histones is subject to cell type specific regulation.

Group 4 contained two RNA helicases, DDX21 and DDX50 and the nucleolar RNA binding protein EBP2. Group 4 also contained the transcriptional regulators TAF5 and TIF1B, as well as CHD7. While CHD7 has not been shown to be associated with linker histones, these results suggest that it may function in a manner analogous to CHD8.

Only two proteins were found to be exclusive binding partners of H1x (group 6). These were ubiquitin carboxyl-terminal hydrolase 15 (USP15) and insulin-like growth factor 2 mRNA-binding protein 3 (IGF2BP3). USP15 is a deubiquitinating enzyme that can bind to ubiquitinated H2A/H2B dimers, and removes ubiquitin from ubiquitinated H2B that are not in nucleosomes [50]. IGFBP3 belongs to a family of three IGF-II mRNA-binding proteins that can bind to the 5′ UTR of the insulin-like growth factor II leader 3 mRNA and regulate the translation of insulin-like growth factor II during late mammalian development [51]. Interactions between USP15 and IGFBP3 and linker histones have not been reported previously.

Group 6 is an eclectic collection of proteins that co-purified with both H1.1 and H1x. This group includes the RNA binding proteins SRSF3 and SRSF7, the zinc finger protein ZNF16 and the anti-microbial protein dermcidin.

### Interactions between nucleoprotein Tpr and H1 variants

**Fig. 4:**
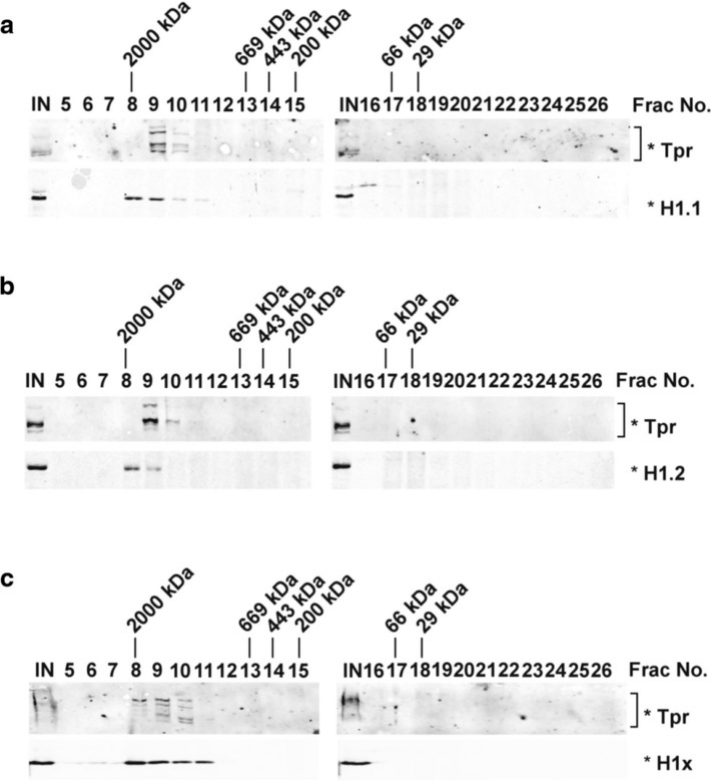
Tpr Co-Elutes with H1 Variants on Size Exclusion Column. Soluble whole cell extracts of U2OS cells expressing 6 × His - tagged H1.1 (**a**), H1.2 (**b**) or H1x (**c**) were resolved by gel filtration chromatography (Superose 6). Fractions (indicated by numbers at top of gels) were precipitated with 20 % *w/v* TCA, then resolved by SDS-PAGE gel, and visualized by immunoblotting with anti-Tpr or anti-His antibodies

The proteomics data clearly demonstrate that linker histones participate in a complex set of interactions that display varying degrees of specificity for the H1 variants. To explore in more detail the distinction between replication-dependent (H1.1 and H1.2) and replication-independent (H1x) H1 variants, we examined whether the specificity observed for replication-dependent H1.1 and H1.2 displayed by Tpr was a reflection of a specific in vivo connection between Tpr and replication-dependent linker histones. Unlike the PAF-1 complex and CHD8, which were also identified in the H1.1 and H1.2 complexes but not in the H1x complex (Table 1), Tpr has not been previously shown to interact with linker histones. To confirm the potential for interactions between Tpr and H1 variants in vivo, we resolved soluble whole cell extracts of U2OS cells expressing H1.1, H1.2 or H1x by size exclusion chromatography (Fig. 4). In all three cases, the peaks of H1 variants overlapped with the Tpr peak in a very high molecular weight complex (Fraction 9–10 in Fig. 4a, Fraction 9 in Fig. 4b, and Fraction 8–10 in Fig. 4c). These large complexes are likely due to other proteins associated with Tpr and/or H1 variants through direct or indirect interactions. The overlapping elution profiles of the linker histones and Tpr is consistent with the possibility that Tpr can form complexes with the linker histones in vivo but does not provide biochemical evidence for specific association of Tpr with the replication-dependent linker histones. Recprical co-immunopreciptations were also attempted to obtain additional evidence for for the specific interactions between Tpr and linker histones. However, currently available anti-Tpr antibodies were efficient for the immunopreciptation of Tpr and its associated proteins.

### Tpr knockdown specifically reduces replication-dependent H1 variant levels via protein degradation

**Fig. 5:**
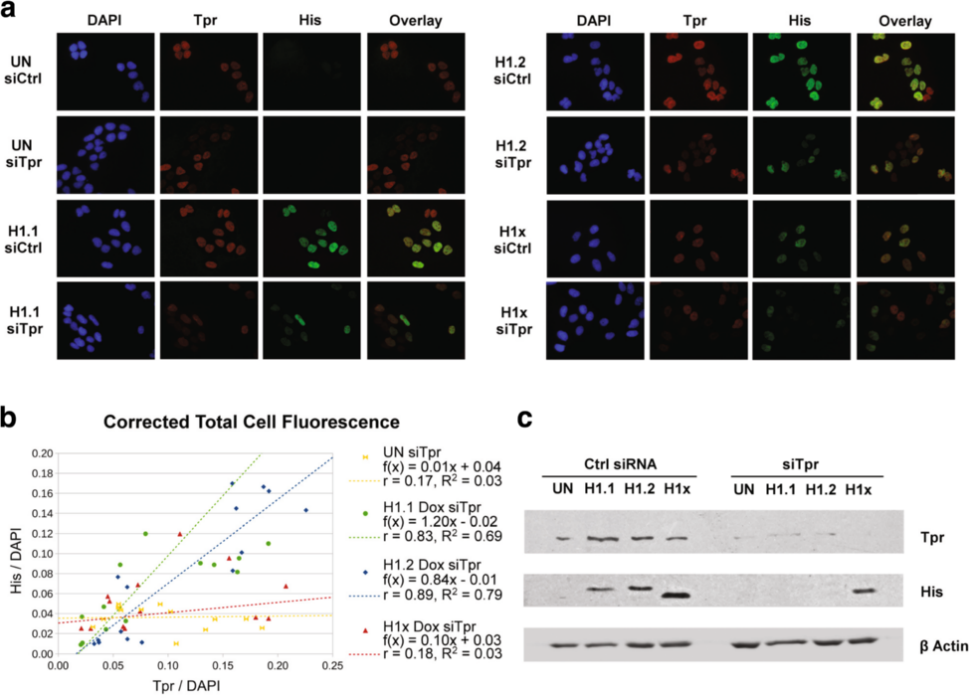
Tpr Knockdown Specifically Reduces Replication-dependent H1 Variant H1.1 and H1.2 Levels. **a** Untransfected (UN) U2OS Tet/On cells or U2OS Tet/On cells expressing H1 variants (H1.1, H1.2 and H1x) were treated with either control siRNA or siRNA targeting protein Tpr for 72 h. The cells were simultaneously stained with DAPI (*blue*), anti-Tpr (*Red*), and anti-His (*Green*). Overlay of anti-Tpr and anti-His staining was shown for comparison. **b** Scatter plot of quantified fluorescence intensities from Fig. 5a. Quantification and regression analysis was performed as described in Experimental Procedures. **c** Untransfected (UN) U2OS Tet/On cells or U2OS Tet/On cells expressing H1 variants (H1.1, H1.2 or H1x) were treated with either control siRNA or siRNA targeting protein Tpr for 72 h. 20 μg whole cell extracts of each sample were resolved on SDS-PAGE gel, and visualized by immunoblotting with antibodies targeting proteins indicated on the right

To determine whether the observed interactions between Tpr and replication-dependent linker histones are functionally relevant, we treated U2OS cells expressing 6 × His-tagged H1.1, H1.2 or H1x with Tpr-targeting siRNA in order to knockdown the expression of Tpr proteins. Surprisingly, we found that siTpr treated cells, which had significantly reduced Tpr immunofluorescence signals, also showed reduced immunofluorescence signals of 6 × His-tagged replication-dependent variants H1.1 and H1.2, but not replication-independent variant H1x (Fig. 5a). In both control siRNA and Tpr siRNA treated cells, all three H1 variants were localized within the nucleus, indicating that the loss of Tpr did not disrupt the nuclear distribution of histone H1. The fluorescence intensities of the signals for Tpr and the H1 variants, relative to the signal for DAPI, were quantitated and plotted (Fig. 5b). Based on a linear regression analysis of the data there was a clear positive correlation between the level of Tpr and the levels of histone H1.1 and H1.2 (*r* = 0.83 and 0.89, respectively) while there was no correlation between the level of Tpr and the level of H1x (*r* = 0.17). Consistent with the immunofluorescence data, we also observed reduced H1.1 and H1.2 protein levels in Tpr siRNA treated cells on western blots; however H1x protein levels were not affected (Fig. 5c).

**Fig. 6:**
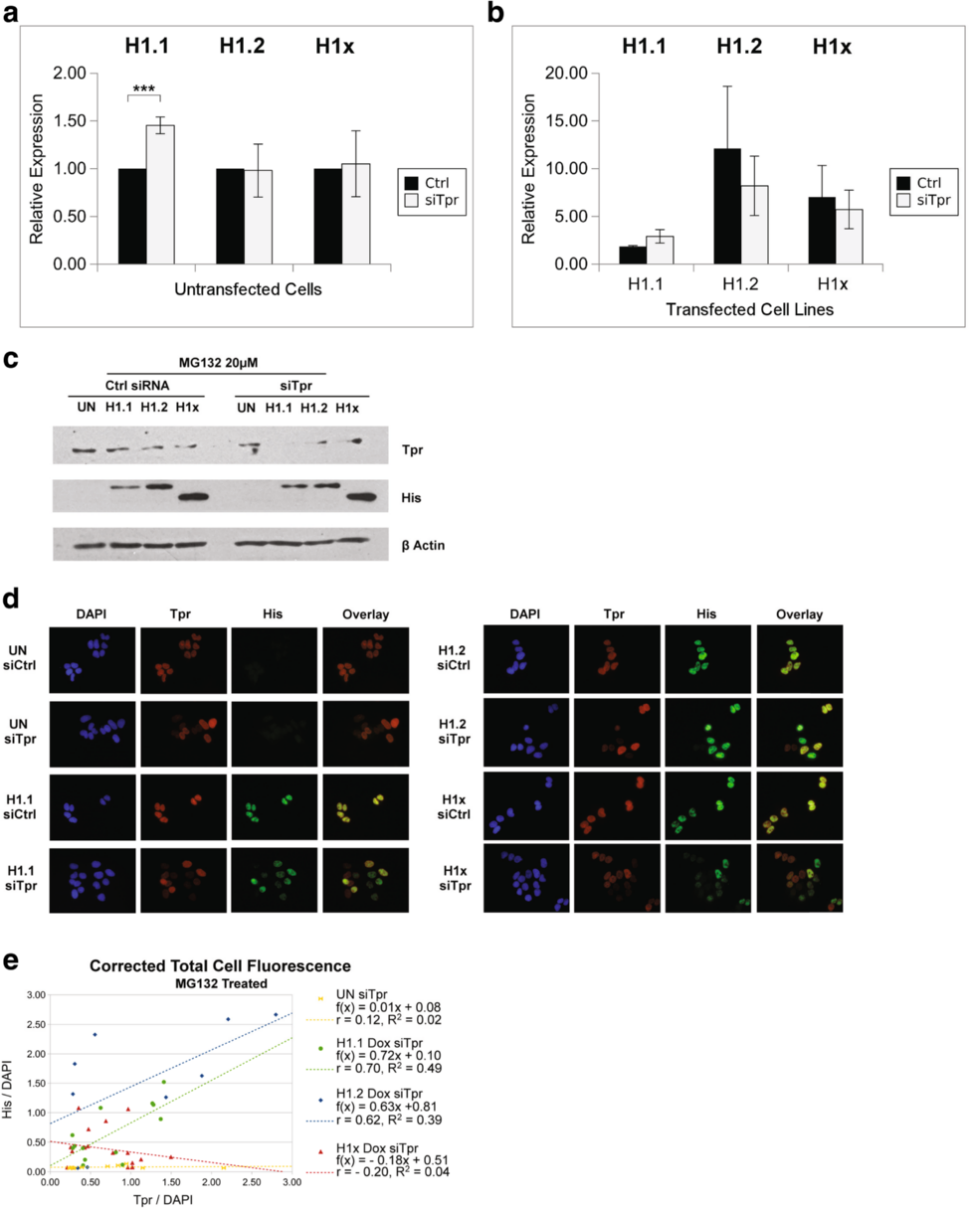
Tpr Stabilizes Replication-dependent H1 Variant H1.1 and H1.2 by Preventing Protein Degradation. **a**–**b** mRNA levels of H1.1, H1.2 or H1x were quantified by quantitative Real-Time PCR in untransfected U2OS Tet/On cells (**a**) or U2OS Tet/On cells expressing H1 variants H1.1, H1.2 or H1x (**b**) treated with either control siRNA or siRNA targeting Tpr for 48 h. Expression levels of histone H1 genes were normalized relative to levels of GAPDH. Each experiment was completed on three biological replicates where each biological replicate was obtained in triplicate, and the mean of these values was used for further analysis. Statistical analysis was carried out using unpaired Student’s t-test: *p* ≤ 0.05 (*), *p* ≤ 0.01 (**), and *p* ≤ 0.001 (***). The data are presented as means ± standard deviations (*n* = 3). **c** Untransfected (UN) U2OS Tet/On cells or U2OS Tet/On cells expressing H1 variants (H1.1, H1.2 or H1x) were treated with either control siRNA or siRNA targeting protein Tpr for 72 h. For the last 3 h of siRNA transfection, 20 μM of MG132 was added to the media. 20 μg whole cell extracts of each sample were resolved on SDS-PAGE gel, and visualized by immunoblotting with antibodies targeting proteins indicated on the right. **d** Untransfected (UN) U2OS Tet/On cells or U2OS Tet/On cells expressing H1 variants (H1.1, H1.2 or H1x) were treated with either control siRNA or siRNA targeting protein Tpr for 72 h. For the last 3 h of siRNA transfection, 20 μM of MG132 was added to the media. Treated cells were then simultaneously stained with DAPI (*blue*), Tpr (*Red*), and 6 × His-tag (*Green*). Overlay of anti-Tpr and anti-His staining was shown for comparison. **e** Scatter plot of quantified fluorescence intensities from Fig. 6d. Quantification and regression analysis was performed as described in Experimental Procedures

The reduction in H1.1 and H1.2 levels in Tpr knockdown cells could have been a result of a decrease in mRNA abundance or by protein destabilization. In order to investigate the effect of Tpr depletion on histone H1 gene expression, we performed quantitative real-time PCR analysis on the mRNA levels of H1.1, H1.2, and H1x in U2OS cells treated with either control siRNA or Tpr siRNA. Tpr knockdown did not cause any significant decrease in the mRNA levels of any of the three H1 variants (Fig. 6a and b).

To test whether Tpr is required for histone H1.1 and H1.2 protein stability, we treated U2OS cells with MG132, which is a cell permeable proteasome inhibitor. In the presence of MG132, H1.1 and H1.2 protein levels were restored in Tpr siRNA treated cells to levels comparable to those of control siRNA treated cells (Fig. 6c). Immunofluorescence microscopy of MG132 treated cells also confirmed this rescue of H1.1 and H1.2 protein levels (Fig. 6d). Quantitation of the immunofluorescence data indicated a clear decrease in the correlation of the levels of Tpr and histones H1.1 and H1.2 with an increase in the number of cells with a low level of Tpr that maintain a high level of the replication-dependent linker histones (Fig. 6e). It is important to note that in MG132 treated cells all H1 variants were still localized within the nucleus, indicating that loss of Tpr did not cause replication-dependent H1 proteins to accumulate in the cytosol suggesting that Tpr is not required for the nuclear import of replication-dependent linker histones. These results suggest that the specific interaction between Tpr and replication-dependent histone H1 variants detected biochemically is a reflection of a specific in vivo interaction. Further, the interaction between Tpr and histones H1.1 and H1.2 is critical to maintain the proper abundance of these linker histones.

## Discussion

There is accumulated evidence indicating functional differentiation among linker histone variants. The abundance of H1 variants fluctuates in diverse cell types and at development stages [6, 52]. Their ability to condense chromatin also varies (reviewed in [53]). In addition, the H1 variants can engage in a wide range of different protein-protein interactions (reviewed in [16, 54]). Several previous proteomic studies focused on the characterization of the linker histone variants H1.0 and H1.2 interaction networks, however the comparison of replication-dependent and replication-independent H1 variants is still lacking [14, 15, 37]. In order to understand how replication-dependent and replication-independent H1 variants carry out differentiated functions through protein-protein interactions, we purified protein complexes containing 6 × His-tagged replication-dependent H1 variants H1.1 and H1.2, or replication-independent variant H1x, and identified their binding partners using mass spectrometric analysis.

Broadly speaking, our proteomic characterization of linker histones binding proteins identifies four groups of proteins that interact with linker histones. We used soluble whole cell extracts as the starting material for the H1 purifications to facilitate the identification of histone chaperones that would regulate the transport and assembly of the linker histones. Indeed, a significant fraction of the H1 interacting proteins are previously known histone-binding proteins. While many of these proteins have primarily been characterized in the context their functions as core histone chaperones, it will be interesting to determine how they also impact linker histone dynamics.

The well-characterized function of linker histones in the regulation of transcription is reflected in the abundance of transcription factors identified as linker histone binding proteins. The observation that some transcription factors are highly specific for a single histone H1 variant suggests that some of the unique functions of the linker histones may be mediated by protein-protein interactions that regulate specific subsets of genes.

The other two groups of proteins were related to RNA biology. The largest groups of interacting factors were ribosomal proteins. The interaction of linker histones with ribosomes has been observed both in vitro and in vivo and has been implicated in transcriptional repression [14, 15, 17]. We also identified a number of proteins involved in RNA metabolism including RNA binding proteins and RNA helicases. Surprisingly, multiple members of the WTAP complex, which is required for a significant fraction of the N^6^-methyladenosine modification in the cell, as well as the N^6^-methyladenosine reader protein YTDC1 were associated with all three H1 variants [28]. Together, these results suggest the intriguing possibility that linker histones may provide an important link between chromatin structure and RNA metabolism.

Surprisingly, NASP (nuclear antigenic sperm protein) was not found to associate with any of the histone H1 variants in the present study and has not been identified in the recent proteomic analyses of in vivo linker histone complexes [16, 26, 37]. NASP was one of the first proteins proposed to be a linker histone chaperone and, in fact, can bind linker histones with nM affinity in vitro [55–57]. The inability to identify NASP as linker histone-associated protein in multiple studies suggests a number of possibilities. NASP may not interact with linker histones to a significant extent despite the demonstrated in vitro affinity. Alternatively, NASP may interact in vivo with only a subset of the linker histone variants that have not yet been examined by proteomic methods.

In comparing the proteins that co-purified with each of the H1 variants, we found that the three H1 variants had shared binding partners that were identified in earlier studies, such as nucleophosmin, nucleolin and FACT complex subunits SPT16 and SSRP1, nucleolin, nucleophosmin (NPM1) and Y box-binding proteins YB1, YB2 and YB3. These H1-associating proteins may contribute to the common functions of H1 variants. Importantly, we identified shared components found in the replication-dependent H1 variant complexes that were not associated replication-independent H1 variants. These factors include the PAF1 complex, CHD8 and nucleoprotein Tpr. The recent study from Kim and colleagues demonstrated that the PAF1 complex is not associated with histone H1.0 consistent with the specificity of this complex for replication-dependent linker histones [37]. These exclusive protein-protein interactions may be crucial for mediating cell cycle-dependent functions of H1 variants.

The interactions of the PAF-1 complex and CHD8 with linker histones have demonstrated functions in transcriptional regulation [37–39]. However, a link between Tpr and linker histones has not previously been identified. Our analysis of Tpr siRNA knockdown cells supports the proteomic data that indicated that Tpr engages in specific interactions as Tpr knockdown reduced the levels of replication-dependent H1 variants H1.1 and H1.2, but not replication-dependent H1 variant H1x. Future studies will determine whether Tpr also interacts with the remaining replication-dependent linker histones, H1.3, H1.4 and H1.5 or, whether, like the PAF1 complex, it is specific for H1.1 and H1.2.

While Tpr is required for the stability of histones H1.1 and H1.2, the functional significance of their interaction is not clear. As Tpr is a component of the nuclear pore complex, the most straightforward model is that Tpr is specifically involved in the nuclear import of replication-dependent linker histones. This model predicts that if the levels of H1.1 and H1.2 were restored in cells lacking Tpr that the histones would accumulate in the cytoplasm. However, when the Tpr siRNA knockdown was performed in the presence of the proteasome inhibitor MG132, we observed no cytoplasmic accumulation of H1.1 and H1.2.

A second possibility is that Tpr can function as a histone chaperone that participates in the targeting of replication-dependent linker histones to sites of chromatin assembly. Indeed, Tpr contains a region nears the COOH-terminus that is highly enriched in aspartic acid and glutamic acid as is observed in many histone chaperones. In addition, a recent proteomic analysis of nascent chromatin structure identified Tpr as a chromatin-associated protein [58].

Another potential function for the interaction of Tpr with histones H1.1 and H1.2 may be related to involvement of Tpr in the formation of heterochromatin exclusion zones (HEZs) around nuclear pores. While heterochromatin localizes near the nuclear envelope, regions near the nuclear pores are devoid of heterochromatin through the formation of Tpr-dependent HEZs [41]. The interaction between Tpr and replication-dependent H1 variants may be important for the maintenance of HEZs. Tpr may stabilize and preserve H1 variants with minimal DNA compaction abilities, such as H1.1 and H1.2, which establishes a basal level of chromatin packaging around HEZs, while keeping out H1 variants that promote strong condensation, such as H1x. To test this hypothesis, it would be interesting to investigate the interactions between Tpr and other H1 variants with higher affinity and/or higher capacity for the compaction of nucleosomes, such as H1.3 and H1.4 (Reviewed in [53]).

## Conclusions

We have isolated and analyzed the soluble forms of the replication-dependent linker histones H1.1 and H1.2 and the replication-independent linker histone H1x. These linker histones interact with both a common group of proteins and with variant-specific interacting proteins. One protein that is specifically associated with H1.1 and H1.2 is nuclear pore complex protein Tpr. Tpr was not required for the nuclear import of histones H1.1 and H1.2. However, knockdown of Tpr expression resulted in a specific decrease in protein stability of the replication-dependent linker histones.

## Methods

### Antibodies and other materials

Sources of antibody employed are as follows: anti-His.M8 from Thermo Fisher Scientific was used for western blots; anti-His_6_ from Roche was used for immunofluorescence microscopy; anti-Tpr from Abcam. Control siRNA (sc-37007) and siRNA targeting Tpr (sc-45343) was purchased from Santa Cruz Biotech.

### Plasmid construction

The human Ultimate ORF of histone H1.1 (IOH35288), H1.2 (IOH5275), and H1x (IOH3417) (Thermo Fisher Scientific) were inserted into mammalian expression vector pT-Rex-DEST31 (Thermo Fisher Scientific), which adds a 6 × His-tag at the NH_2_-terminus. The resulting constructs were transferred into DH10B *E. coli* strain for production.

### Cell culture and transfections

Tetracycline-inducible human osteosarcoma cell line U2OS (kind gift from Dr. Dan Schoenberg) was grown in McCoy’s 5A media supplemented with 10 % (*v/v*) fetal bovine serum, 50 U/ml penicillin, 50 mg/ml streptomycin, and 1 % L-glutamate (Sigma) at 37 °C in 5 % CO_2_ supply. The U2OS cells were transfected with pT-Rex-DEST31 plasmids carrying H1.1, H1.2 or H1x using X-tremeGENE HP DNA transfection reagent (Roche) following the manufacturer’s instruction. Transfected clones were selected against 200 mg/ml G418 and 10 mg/ml blasticidin (Gibco, Thermo Fisher Scientific). Stable clones were induced by 1 μg/ml doxycycline (Sigma) for 48 h. The expression of 6 × His-tagged H1 variants was confirmed by western blot.

### Cell fractionation

Cytosolic and nuclear extracts were prepared from U2OS cells as previously described [59]. The resulting extracts were resolved on SDS-PAGE gels and visualized on western blots.

### Histone extractions

Total histones were prepared from U2OS cells as previously described [60]. The resulting histone pellets were resuspended in DN (300) buffer (300 mM NaCl, 25 mM Tris pH 7.0, 0.1 mM EDTA, 10 % glycerol, and 0.5 mM phenyl methyl sulfonate fluoride), and resolved on 18 % SDS PAGE gels. Linker histones isolated from chicken erythrocyte nuclei were also loaded in the gels as controls [56].

### Micrococcal nuclease digestion assays

1 × 10^7^ U2OS cells were collected and washed with cold PBS, and incubated in lysis buffer (300 mM HEPES pH7.5, 60 mM KCl, 300 mM sucrose, 5 mM K_2_HPO_4_, 5 mM MgCl_2_, 2 mM EDTA, and 0.5 % Triton X-100) for 5 min on ice. The cells were then broken with 7 gentle strokes in a type B Dounce homogenizer. Nuclei were pelleted by centrifuging at 120 × g for 10 min. The pelleted nuclei were washed with 1 ml MNase digestion buffer (10 mM Tris pH7.5, 15 mM NaCl, 1 mM CaCl_2_, 60 mM KCl, and 0.2 mM phenyl methyl sulfonate fluoride), resuspended in 1 ml MNase digestion buffer, and digested with 0.2 U/ml (Sigma units) of MNase at 37 °C for various lengths of time. Digestions were stopped with 10 mM EDTA followed by incubation with 0.1 mg/ml proteinase K at 37 °C for 5 min. The resulting DNA samples were isolated using phenol extraction and ethanol precipitation, and resolved on 1 % agarose gel [61].

### Protein expression and purification

U2OS cells with 6 × His-tagged H1 inserts were induced by 1 μg/ml doxycycline (Sigma). After 48 h, cells were harvested, washed with PBS, and lysed with NP40 lysis buffer (150 mM NaCl, 50 mM Tris pH7.5, 10 % Glycerol, 0.75 % NP40) containing EDTA-free protease inhibitor cocktail (Roche) and 0.5 mM phenyl methyl sulfonate fluoride. The resulting whole cell extracts were applied to a Mono Q 5/50 GL column (GE Healthcare Life Sciences). Proteins were eluted with a NaCl gradient from 50 mM to 500 mM in 15 column volumes, then 1 M for 5 column volumes. Fractions containing eluted 6 × His-tagged H1 variants were pooled, desalted on desalting columns (GE Healthcare Life Sciences), and then applied to an HiTrap Chelating HP Column (1 ml, GE Healthcare Life Sciences) charged with Ni^2+^. The column was then washed extensively with NP40 lysis buffer containing 50 mM imidazole. Bound proteins were eluted with NP40 lysis buffer containing 500 mM imidazole. Fractions were collected and resolved on western blots.

### Mass spectrometry

Tryptic peptides were desalted online using a μ-precolumn (PepMap100, C18, 5 μm, 100 Å, 0.3 x 50 mm, ThermoFisher, Waltham, MA, USA) and separated based on hydrophobicity at a flow rate of 2 μL/min (C18Aq, 5 μm, 300 Å 0.2 × 150 mm, Michrom Bioresources Inc., Auburn, CA, USA). Peptides were introduced into a ThermoFisher LTQ Orbitrap XL mass spectrometer (ThermoFisher, Waltham, MA, USA) with the aid of micro/nanospray ionization source (Michrom Bioresources Inc., Auburn, CA, USA). Mobile phase A (MPA) consisted of HPLC water with 0.1 % *v/v* formic acid (J.T. Baker, Center Valley, PA, USA) and mobile phase B (MPB) consisted of ACN (EMD Millipore, Billerica, MA, USA) with 0.1 % v/v formic acid. The HPLC gradient was ramped from 2 to 40 % MPB from 5 to 155 min, followed by a high organic wash and column equilibration period. The heated capillary temperature and electrospray voltage were set to 175 °C and 2.0 kV. The top 5 molecular ions (2+/3+ charges) were selected for CID fragmentation in a data-dependent fashion. Molecular ions were analyzed in the Orbitrap (AGC:1000 K ions; 60 K mass resolution; 300 ms max injection time, 1 microscan, precursor scan enabled). Molecular ions were selected and subjected to CID fragmentation in the linear ion trap (NCE: 35 %, AGC: 10 K ions; 300 ms max injection time). Dynamic exclusion parameters were enabled (30s ± 25 ppm, repeat count:1). Protein identifications were determined using the MassMatrix search engine (v 2.4.2) and the complete, reviewed Swiss-Prot entries from UniProt (taxon:9606, 20,258 entries, July 2013) [62–65]. Search parameters included a maximum of two trypsin missed cleavages, molecular ion tolerance of ±20 ppm and a fragment ion tolerance of ±0.8 Da. The false discovery rate (FDR) was estimated using a target-decoy strategy. Final protein identifications and spectral counts were harmonized using an in-house python application [66]. Protein identification from this harmonized protein list, were retained based on an FDR threshold of 5 % where each protein consisted of at least two unique peptide matches.

### Size-exclusion chromatography of H1 complexes

Whole cell extracts of U2OS cells expressing 6 × His-tagged H1 variants were applied to a Superose 6 10/300 GL column (GE Healthcare Life Sciences). The column was washed with 1.5 column volumes of NP40 lysis buffer. Proteins in each collected fraction were precipitated with 20 % *w/v* TCA, then resolved and visualized on western blots.

### Immunofluorescence microscopy

U2OS cells seeded on cover slips were fixed in 4 % paraformaldehyde for 20 min at room temperature and then washed with PBS. The washed cells were permeabilized with 0.5 % Triton X-100 in PBS for 15 min, then washed with PBS three times, and blocked with blocking buffer (1 % bovine serum albumin and 0.3 % Triton X-100 in PBS) for 1 h. After blocking, the cells were incubated with primary antibodies diluted in blocking buffer overnight followed by three washes in PBST, and incubated with secondary antibodies for 1 h. Then the cover slips with cells were washed three times in PBS, and mounted onto microscope slides with Vectashield mounting serum containing DAPI (Vector Laboratories). The slides were examined using Axioskop 40 microscope. Immunofluorescence pictures were taken with an Axiocam HRC camera and processed in Zen 2 Pro software.

### Quantification and analysis of immunofluorescence

Corrected total cell fluorescence (CTCF) was quantified using ImageJ as previously described [67]. CTCF of anti-Tpr and anti-His_6_ staining were standardized against CTCF of DAPI staining. Regression analysis was carried out using Microsoft Excel built-in statistical functions.

### Quantitative real-time PCR analysis

RNAs were extracted from U2OS cells using Trizol reagent (Thermo Fisher Scientific) following the manufacturer’s instructions. The resulting RNAs were used to prepare cDNAs using High Capacity cDNA Reverse Transcription Kits (Applied Biosystems) following the manufacturer's instruction. Levels of gene expression were measured using TaqMan Gene Expression Assays (Applied Biosystems, Assay IDs as follows: H1.1 – Hs00271225_s1, H1.2 – Hs00271185_s1, H1x – Hs00366688_s1, and GAPDH – Hs02758991_g1), TagMan Universal PCR Master Mix (Applied Biosystems) and the ABI 7300 sequence detector as previously described [68]. Expression levels of histone H1 genes were normalized relative to levels of GAPDH. Each experiment was completed on three biological replicates where each biological replicate was obtained in triplicate, and the mean of these values was used for further analysis. Statistical analysis was carried out using unpaired Student’s t-test: *p* ≤ 0.05 (*), *p* ≤ 0.01(**), and *p* ≤ 0.001(***).

## Additional file

Additional file 1: Table S1. Protein identification report for H1 variant associating proteins. The number of unique peptides determined by mass spectrometry are given for each protein. Mock purification sample from U2OS Tet/On cells without H1 vector insert was used as negative control. Two technical replicates were analyzed for each H1 variant sample. Four technical replicates were analyzed for the negative control. (XLSX 67 kb)
